# Supplementary material for: Discovery, total syntheses and potent anti-inflammatory activity of pyrrolinone-fused benzoazepine alkaloids Asperazepanones A and B from Aspergillus candidus
Source: Commun Chem. 2022 Jul 6;5:80. doi: 10.1038/s42004-022-00696-2 (PMC9814288; doi:10.1038/s42004-022-00696-2)
Supplement: Supplementary file 1 — Supplementary Information [file 42004_2022_696_MOESM1_ESM.docx]

**Supplementary Information**

Discovery, total syntheses and potent anti-inflammatory activity of pyrrolinone-fused benzoazepine alkaloids Asperazepanones A and B from *Aspergillus candidus*

Li Xu^†, ‡, #^, Feng-Wei Guo^†, ‡, #^, Xue-Qing Zhang^†, #^, Tian-Yi Zhou^†^, Chao-Jie Wang^†^, Mei-Yan Wei^†, ┴^, Yu-Cheng Gu^+^, Chang-Yun Wang^†, ‡^, and Chang-Lun Shao*^, †, ‡^

^†^ Key Laboratory of Marine Drugs, The Ministry of Education of China, School of Medicine and Pharmacy, Ocean University of China, Qingdao 266003, China.

^‡^ Laboratory for Marine Drugs and Bioproducts, Pilot National Laboratory for Marine Science and Technology (Qingdao), Qingdao 266200, China.

^┴^ College of Food Science and Engineering, Ocean University of China, Qingdao 266003, China.

^+^ Syngenta Jealott’s Hill International Research Centre, Bracknell, Berkshire, RG42 6EY, UK.

**Corresponding Author**

*E-mail: shaochanglun@163.com.

**Author Contributions**

^#^ These authors contributed equally to this work.

**Table of Contents**

**[1. Supplementary Note 1](#_Toc105341897)** [3](#_Toc105341897)

**[1.1. Isolation and Structure Elucidation](#_Toc105341898)** [3](#_Toc105341898)

**[1.1.1. Experimental details](#_Toc105341899)** [3](#_Toc105341899)

**[1.1.2.](#_Toc105341900)****[NMR data](#_Toc105341900)** [5](#_Toc105341900)

**[1.1.3.](#_Toc105341901)****[MS data and IR spectrum](#_Toc105341901)** [6](#_Toc105341901)

**[1.1.4.](#_Toc105341902)****[Crystal structure](#_Toc105341902)** [9](#_Toc105341902)

**[1.1.5.](#_Toc105341903)** **[Differentiation and epimerization property of 1 and 2](#_Toc105341903)** [9](#_Toc105341903)

**[1.1.6. NMR spectra](#_Toc105341904)** [12](#_Toc105341904)

**[2.](#_Toc105341905)****[Supplementary Note 2](#_Toc105341905)** [19](#_Toc105341905)

**[2.1. Total Synthesis](#_Toc105341906)** [19](#_Toc105341906)

**[2.1.1.](#_Toc105341907)****[General methods](#_Toc105341907)** [19](#_Toc105341907)

**[2.1.2.](#_Toc105341908)****[Experimental procedures and characterizations](#_Toc105341908)** [20](#_Toc105341908)

**[2.1.3.](#_Toc105341909)****[Comparison of the](#_Toc105341909) ^[1](#_Toc105341909)^[H NMR and](#_Toc105341909) ^[13](#_Toc105341909)^[C NMR Spectra of Isolated and Synthetic Compounds](#_Toc105341909)** [25](#_Toc105341909)

**[2.1.4.](#_Toc105341910)****[MS data, IR spectrum, Chiral-phase HPLC analysis and Experimental ECD spectra.](#_Toc105341910)** [29](#_Toc105341910)

**[2.1.5.](#_Toc105341911)****[NMR spectra](#_Toc105341911)** [39](#_Toc105341911)

**[3. Supplementary Ntote 3](#_Toc105341912)** [46](#_Toc105341912)

**[3.1. Bioactivity screening](#_Toc105341913)** [46](#_Toc105341913)

**[3.1.1. Materials and reagents](#_Toc105341914)** [46](#_Toc105341914)

**[3.1.2.](#_Toc105341915)****[Cell viability and NO, TNF-α and IL-6 assay](#_Toc105341915)** [46](#_Toc105341915)

**[3.1.3. Effect of compound (+)-2 on the viability and NO production of RAW264.7 cells](#_Toc105341916)** [47](#_Toc105341916)

**[4. Supplementary Reference](#_Toc105341917)** [48](#_Toc105341917)

**1 Supplementary Note 1**

**1.1. Isolation and Structure Elucidation**

**1.1.1. Experimental details**

**Fungal Material.**

The fungal strain *A. candidus* (CHNSCLM-0393) was isolated from a piece of fresh tissue from the inner part of the gorgonian coral *Juncella fragilis* (CHNNS-2016-01), collected from the Nansha Islands coral reef in the South China Sea in April 2016. The strain was identified *A. candidus* according to morphologic traits and molecular identification with the GeneBank (NCBI) accession number MF681708. The strain was deposited at the Key Laboratory of Marine Drugs, the Ministry of Education of China, School of Medicine and Pharmacy, Ocean University of China, Qingdao, China.

**Fermentation and Extraction.**

The fungal strain *A. candidus* (CHNSCLM-0393) was fermented in large-scale under static conditions at room temperature on rice solid medium hydrated with seawater (30 g of natural sea salt (Yangkou saltern), 1L H_2_O) for 8 weeks (one thousand 1000 mL Erlenmeyer flasks, each containing 25 g of rice and 25 mL of seawater). The fermented solid medium was extracted three times with 300 mL EtOAc for each Erlenmeyer flask to give an organic extract (926.9 g).

**Isolation.**

The EtOAc extract was subjected to a macroporous resin column chromatography eluting with ethanol/H_2_O in a gradient from 0: 100 to 0: 100 to obtain five fractions (Fr.1−Fr.5). Fr.2 was separated repeatedly by macroporous resin column chromatography eluting with ethanol/H_2_O in a gradient from 5: 95 to 30: 70 to afford five fractions (Fr.2-1−Fr.2-5). Fr.2-2 was purified on a Sephadex LH-20 eluting with 100% MeOH, then by recrystallization to yield compound **1** (500.0 mg). Fr.2-3 was separated by Sephadex LH-20 eluting with 100% MeOH, then by recrystallization afford compound **2** (800.0 mg). Compound **1** (10.0 mg) was resolved into the corresponding pure enantiomers (+)-**1** (*t*_R_ = 25.2 min, 4.0 mg), (−)-**1** (*t*_R_ = 42.7 min, 4.0 mg) by HPLC using a Chiralpak IC chiral-phase column [5 *μ*m, 4.6 × 150 mm, hexane−ethanol eluent (70: 30), 0.6 mL/min].

**Rapid Extraction and Isolation.**

Three flasks of the fermenation solid medium were extracted with EtOAc for 6h, combined and concentrated under reduced pressure to afford the crude extract. Further, the extract purified by semipreparative HPLC (MeCN-H_2_O with 0.1% TFA, 0‒26 min, 10%–30%, 26‒29 min, 30%–100%, 29‒38 min, 100%, 38‒39 min, 100%–10%, 39‒45 min, 10%; 2 mL/min) to furnish **1** and **2**. **1** and **2** were concentrated under reduced pressure and performed on an enantioselective HPLC (hexane−ethanol eluent (70: 30), 0.6 mL/min) respectively to analyze the natural products and artifacts in one day.

(±)-**Asperazepanone A** (**1**): amorphous solid; **IR** (neat, cm^–1^) ν_max_ 3292, 1624, 1573, 1485, 1384, 1273, 1237, 1200, 792 cm^–1^; **^1^H NMR** (500 MHz, DMSO-*d*_6_) *δ* 10.46 (s, 1H), 9.13 (s, 1H), 7.27 (dd, *J* = 7.9, 1.2 Hz, 1H), 7.22 (s, 1H), 7.06 (dd, *J* = 7.9, 1.2 Hz, 1H), 6.87 (t, *J* = 7.9 Hz, 1H), 5.13 (s, 1H), 4.51 (dd, *J* = 13.1, 2.8 Hz, 1H), 3.03 (dd, *J* = 17.2, 2.8 Hz, 1H), 2.85 (dd, *J* = 17.2, 13.1 Hz, 1H). **^13^C NMR** (125 MHz, DMSO-*d*_6_) *δ* 195.1, 173.9, 162.8, 147.2, 129.5, 125.5, 121.5, 120.9, 118.6, 92.9, 50.2, 47.0. **HRMS (ESI)**: m/z calcd for C_12_H_11_O_3_N_2_ [M+H]^+^ : 231.0764; found: 231.0762.

(−)-**Asperazepanone A** [(−)-10a*R*-**1**]: [α]^25^_D_ –39 (*c* 0.4, MeOH).

(+)-**Asperazepanone A** [(+)-10a*S*-**1**]: [α]^25^_D_ +39 (*c* 0.4, MeOH).

(+)-**Asperazepanone B** [(+)-10a*S*-**2**]: yellow needle; [α]^25^_D_ +130 (*c* 0.2, MeOH); **IR** (neat, cm^–1^) ν_max_ 3380, 1655, 1624, 1571, 1469, 1381, 1300, 1139, 789 cm^–1^;**^1^H NMR** (500 MHz, DMSO-*d*_6_) *δ* 10.26 (s, 1H), 7.21 (s, 1H), 7.15 (overlapped, 1H), 7.12 (overlapped, 1H), 6.88 (dd, *J* = 7.2, 1.6 Hz, 1H), 4.70 (s, 1H), 4.26 (dd, *J* = 11.9, 4.3 Hz, 1H), 3.19 (dd, *J* = 19.0, 4.3 Hz, 1H), 3.16 (s, 3H), 2.50 (dd, *J* = 19.0, 11.9 Hz, 1H). **^13^C NMR** (125 MHz, DMSO-*d*_6_) *δ* 199.8, 173.9, 166.4, 151.6, 135.7, 129.4, 126.8, 120.9, 118.5, 88.8, 51.4, 50.2, 40.3. **HRMS (ESI)**: m/z calcd for C_13_H_13_O_3_N_2_ [M+H]^+^ : 245.0921; found: 245.0917.

Compound **3**: red powder; **IR** (neat, cm^–1^) ν_max_ 3317, 3130, 1713, 1623, 1556, 1543, 1492, 1356, 1280, 1238, 796, 732 cm^–1^; **^1^H NMR** (400 MHz, DMSO-*d*_6_) *δ* 10.89 (s, 1H), 10.29 (s, 1H), 10.00 (s, 1H), 7.06 (d, *J* = 8.0 Hz, 1H), 7.06 (d, *J* = 8.0 Hz, 1H), 6.96 (t, *J* = 8.0 Hz, 1H), 6.31 (s, 1H), 5.79 (s, 1H). **^13^C NMR** (100 MHz, DMSO-*d*_6_) *δ* 183.0, 171.1, 147.5, 146.5, 144.7, 128.6, 123.9, 121.7, 120.8, 117.2, 111.6, 94.5. **HRMS (ESI)**: m/z calcd for C_12_H_9_O_3_N_2_ [M+H]^+^ : 229.0608; found: 229.0606.

**1.1.2. NMR data**

| No. | **1** | |  | **2** | |
| --- | --- | --- | --- | --- | --- |
|  | *δ*_C_, type | *δ*_H_, mult (*J* in Hz) |  | *δ*_C_, type | *δ*_H_, mult (*J* in Hz) |
| 1 |  | 7.22, 1H, s |  |  | 7.21, 1H, s |
| 2 | 173.9 |  |  | 173.9 |  |
| 3 | 92.9 | 5.13, 1H, s |  | 88.8 | 4.70, 1H, s |
| 3a | 162.8 |  |  | 166.4 |  |
| 4a | 129.5 |  |  | 135.7 |  |
| 5 | 147.2 |  |  | 151.6 |  |
| 6 | 118.6 | 7.06, 1H, dd, 7.9, 1.2 |  | 126.8 | 7.15, 1H, overlapped |
| 7 | 121.5 | 6.87, 1H, t, 7.9 |  | 120.9 | 7.12, 1H, overlapped |
| 8 | 120.9 | 7.27, 1H, dd, 7.9, 1.2 |  | 118.5 | 6.88, 1H, dd (7.2, 1.6) |
| 8a | 125.5 |  |  | 129.4 |  |
| 9 | 195.1 |  |  | 199.8 |  |
| 10 | 47.0 | 2.85, 1H, dd, 17.2, 13.1 |  | 50.2 | 2.50, dd (19.0, 11.9) |
|  |  | 3.03, 1H, dd, 17.2, 2.8 |  |  | 3.19, dd (19.0, 4.3) |
| 10a | 50.2 | 4.51, 1H, dd, 13.1, 2.8 |  | 51.4 | 4.26, 1H, dd (11.9, 4.3) |
| 4-NH |  | 9.13, 1H, s |  |  |  |
| 5-OH |  | 10.46, 1H, s |  |  | 10.26, 1H, s |
| 4-CH_3_ |  |  |  | 40.3 | 3.16, 1H, s |
| *^a^* Measured in DMSO-*d*_6_, 500 MHz for ^1^H NMR and 125 MHz for ^13^C NMR. | | | | | |

**Table S1.** ^1^H NMR and ^13^C NMR Data for **1** and **2***^a^*

**1.1.3. MS data and IR spectrum**

**Figure S1.** HRESIMS spectrum of compound **1**.


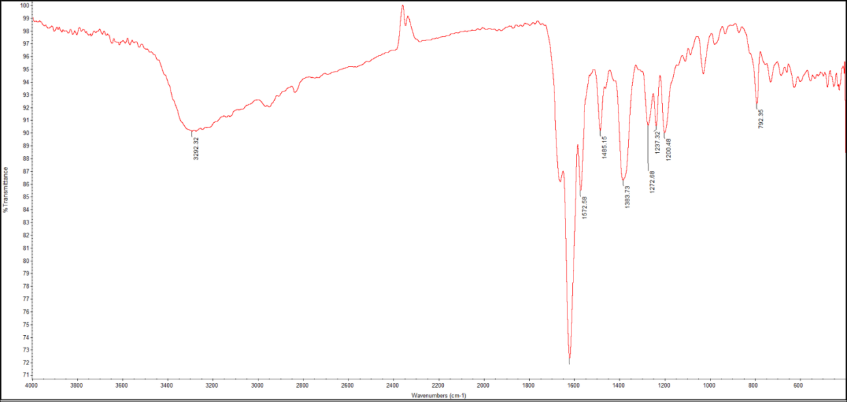


**Figure S2.** IR spectrum of compound **1**.

**Figure S3.** HRESIMS spectrum of compound **2**.


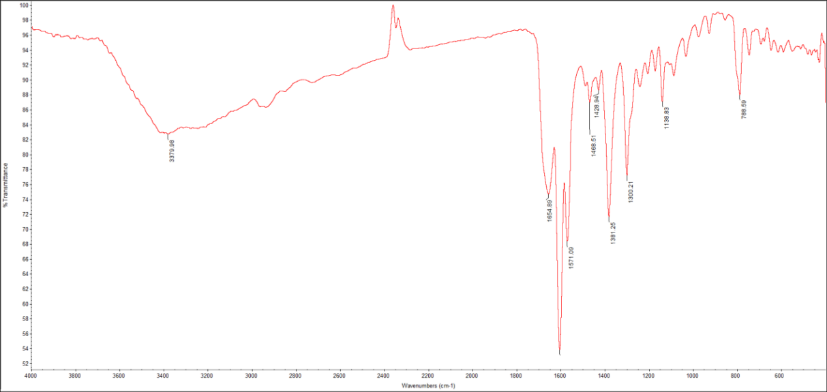


**Figure S4.** IR spectrum of compound **2**.

**Figure S5.** HRESIMS spectrum of compound **3**.


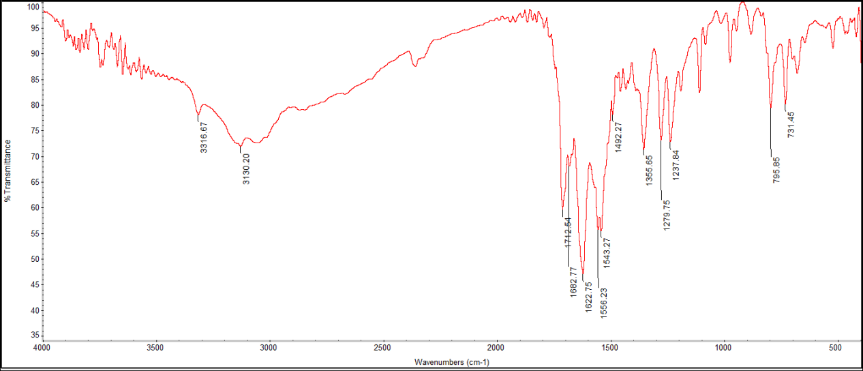


**Figure S6.** IR spectrum of compound **3**.

**1.1.4. Crystal structure**

Colorless crystals of **1** and **2** suitable for X-ray diffraction were obtained from MeOH by slow evaporation. The crystal data were collected at 293 K on an Agilent Gemini Ultra diffractometer with Cu K*α* radiation (*λ* = 1.54184 Å). The structure was solved by direct methods (SHELXS-97) and refined using full-matrix least-squares difference Fourier techniques. All non-hydrogen atoms were refined anisotropically. The crystallographic data for **1** and **2** have been deposited at the Cambridge Crystallographic Data Centre with the deposition numbers 2143832 and 2143833. These data can be obtained, free of charge, on application to the Director, CCDC, 12 Union Road, Cambridge CB21EZ, UK.

*Crystal data for* **1**: C_12_H_10_N_2_O_3_, *M*r = 230.22, monoclinic, space group *P*2_1_/*c* with *a* = 11.1998(13) Å, *b* = 7.3696(14) Å, *c* = 14.552(2) Å, *α* = *γ* = 90°, *β* = 123.710(10), *V* = 999.1(3) Å^3^, *Z* = 4, *D*x = 1.530 mg/m^3^, *μ* (Cu K*α*) = 0.938 mm^−1^, and *F* (000) = 480. Crystal dimensions: 0.12 × 0.11 × 0.11 mm^3^. Independent reflections: 1782 (*R*_int_ = 0.0865). The final *R*_1_ values were 0.0802.

*Crystal data for* **2**: C_13_H_12_N_2_O_3_, *M*r = 244.25, orthorhombic, space group *P*2_1_2_1_ 2_1_ with *a* = 8.1998(4) Å, *b* = 9.4915(4) Å, *c* = 14.9025(8) Å, *α* = *β* = *γ* = 90°, *V* = 1159.84(10) Å^3^, *Z* = 5, *D*x = 1.399 mg/m^3^, *μ* (Cu K*α*) = 0.840 mm^−1^, and *F* (000) = 512. Crystal dimensions: 0.12 × 0.11 × 0.11 mm^3^. Independent reflections: 1608 (*R*_int_ = 0.0343). The final *R*_1_ values were 0.0216.

**1.1.5. Differentiation and epimerization property of 1 and 2**


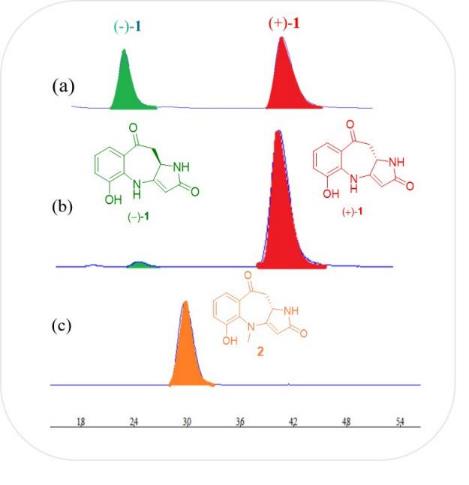


**Figure S7.** Chromatogram of the chiral-phase HPLC of (a) **1** that has been stored for a long time; (b) **1** was quickly separated by fresh extract; (c) **2** was quickly obtained by fresh extract.


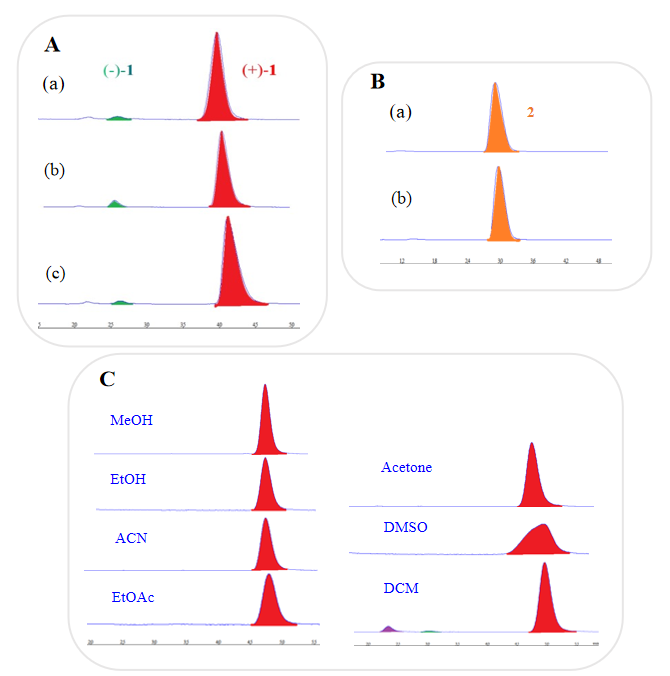


**Figure S8.** Chromatogram of the chiral-phase HPLC analysis of **1** and **2** under different fermentation time, extraction time and solvents.

A: Chromatogram of the chiral-phase HPLC of **1** (a) after 1 month fermentation; (b) after 6 months fermentation; (c) after 1month EtOAc extraction.

B: Chromatogram of the chiral-phase HPLC analysis of **2** (a) after 6 months fermentation; (b) after 1month EtOAc extraction.

C: Chromatogram of the chiral-phase HPLC of **1** soaked under different solvents for one month.


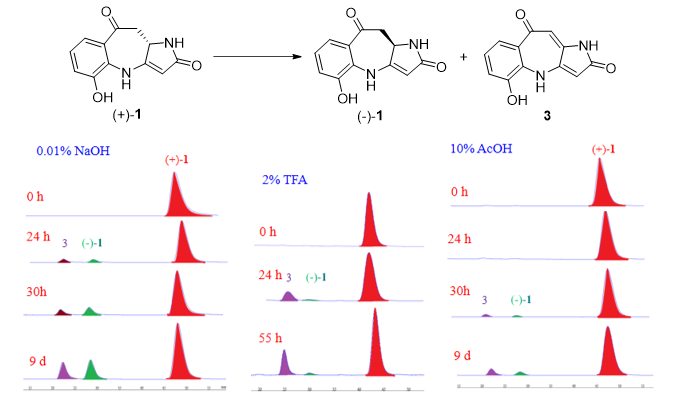


**Figure S9.** The transformation from (+)-10a*S*-**1** to (–)-10a*R*-**1** and **3** in MeOH with different pH levels.


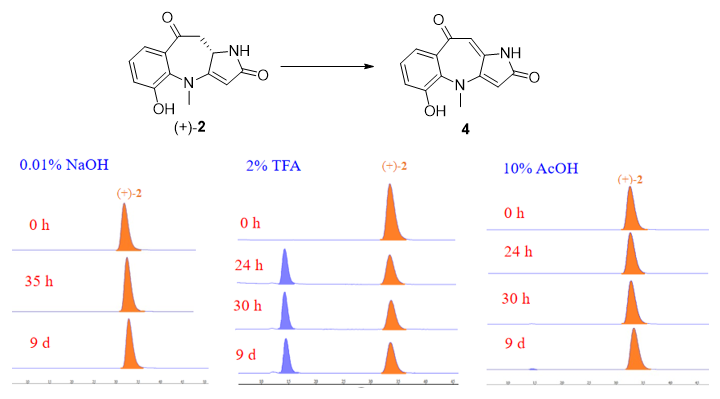


**Figure S10.** Chromatogram of the chiral-phase HPLC analysis of (+)-10a*S*-**2** in MeOH under different pH levels.

**1.1.6. NMR spectra**

^
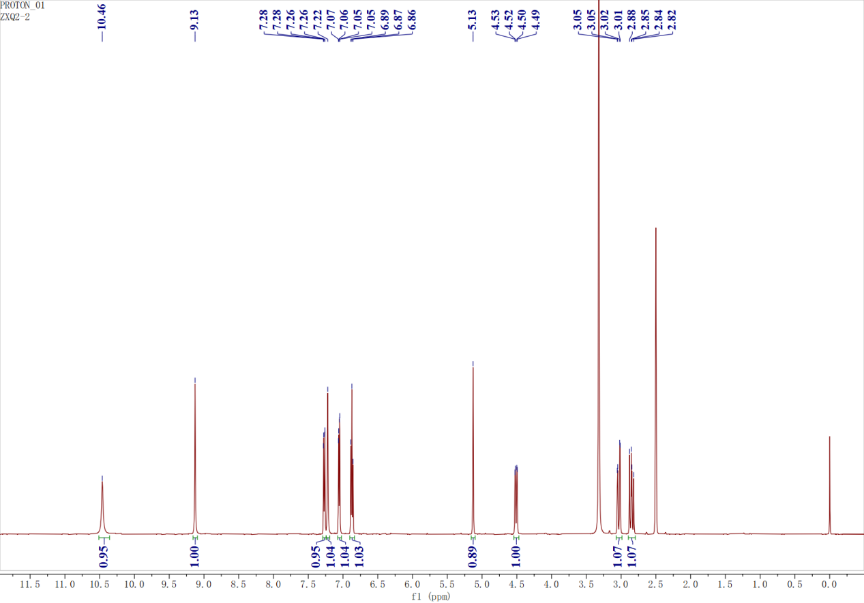
^**Figure S11.** ^1^H NMR spectrum of compound **1** in DMSO-*d*_6_

**
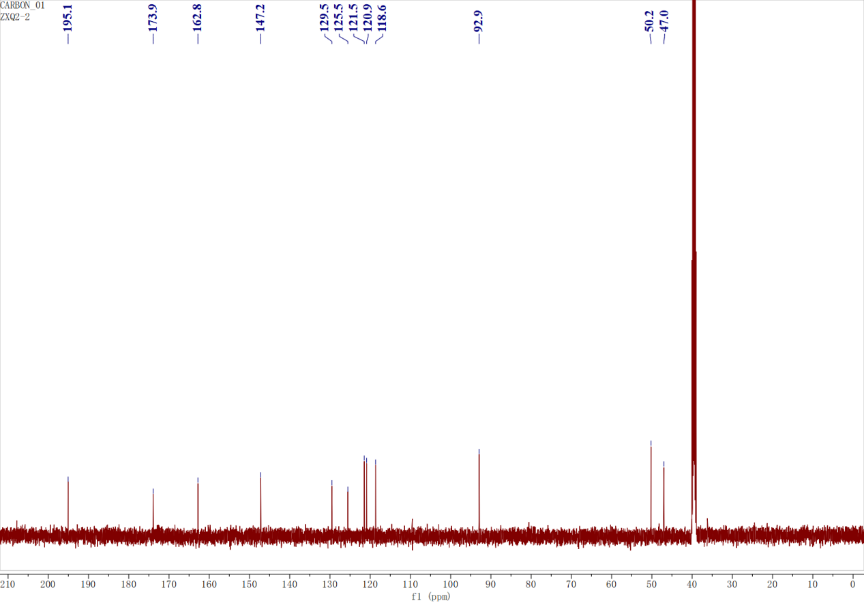
**

**Figure S12.** ^13^C NMR spectrum of compound **1** in DMSO-*d*_6_

_
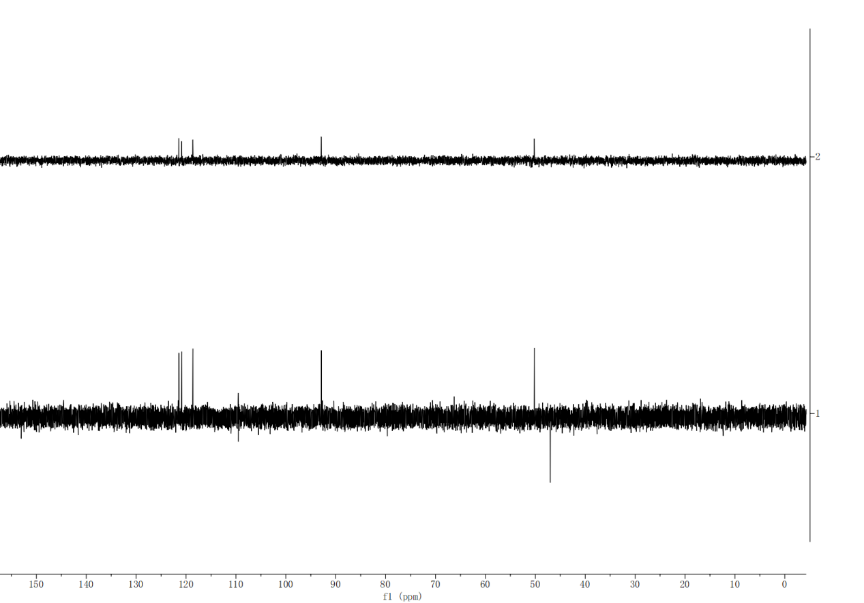
_

**Figure S13.** DEPT spectrum of compound **1** in DMSO-*d*_6_

**
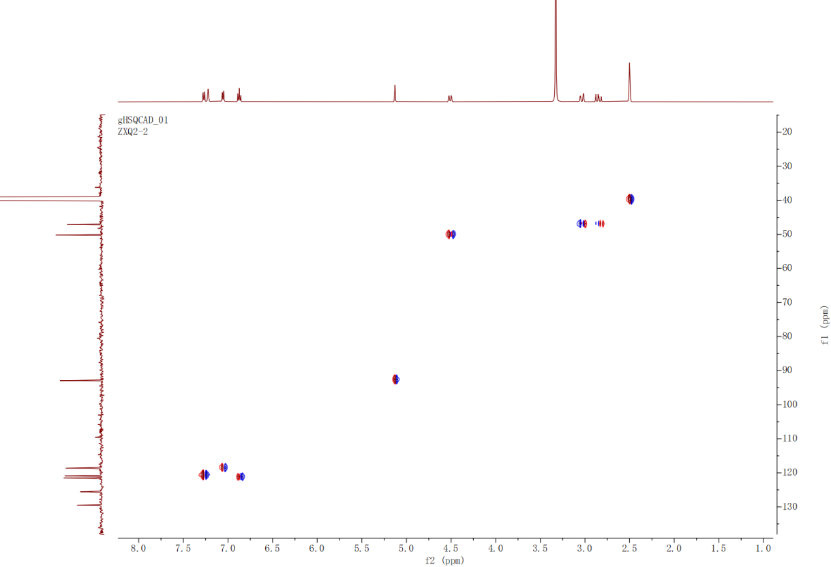
**

**Figure S14.** HSQC spectrum of compound **1** in DMSO-*d*_6_

_
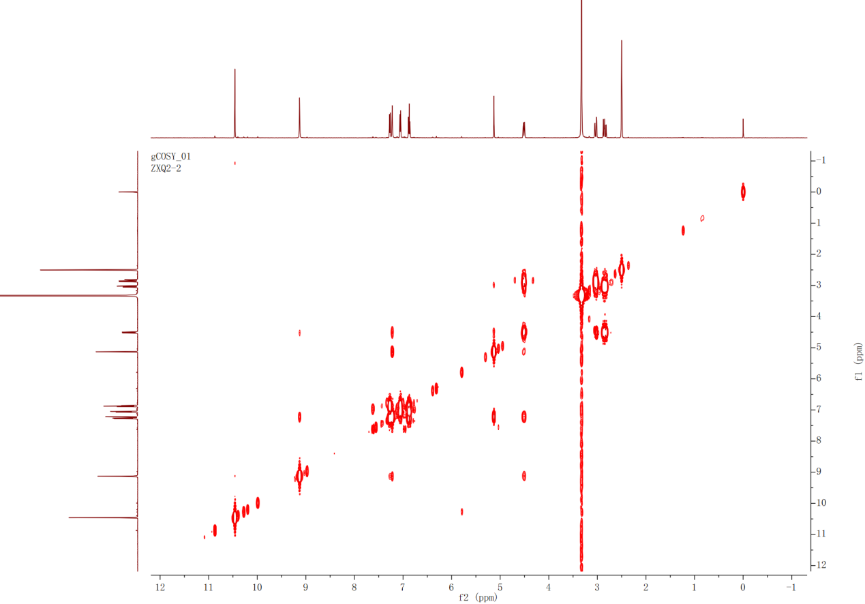
_ **Figure S15.**COSY spectrum of compound **1** in DMSO-*d*_6_

**
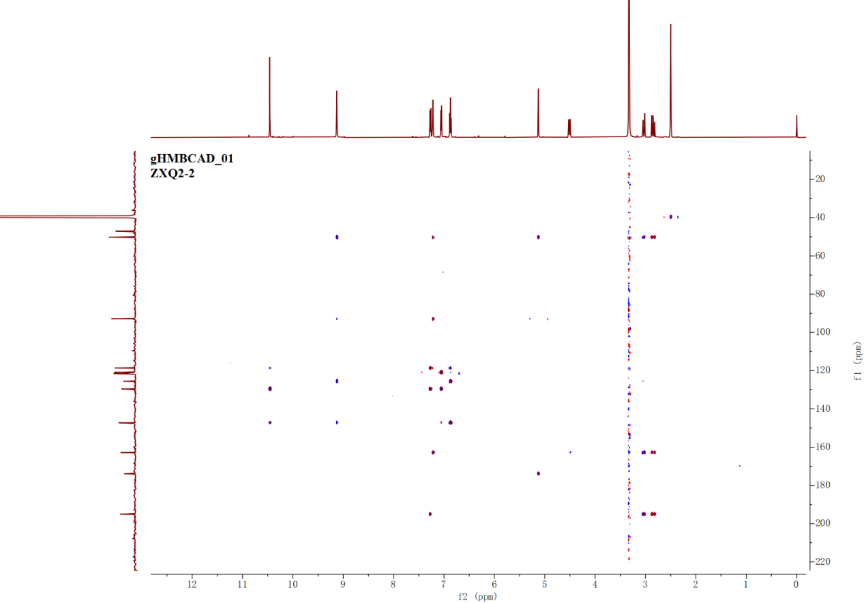
Figure S16.**HMBC spectrum of compound **1** in DMSO-*d*_6_


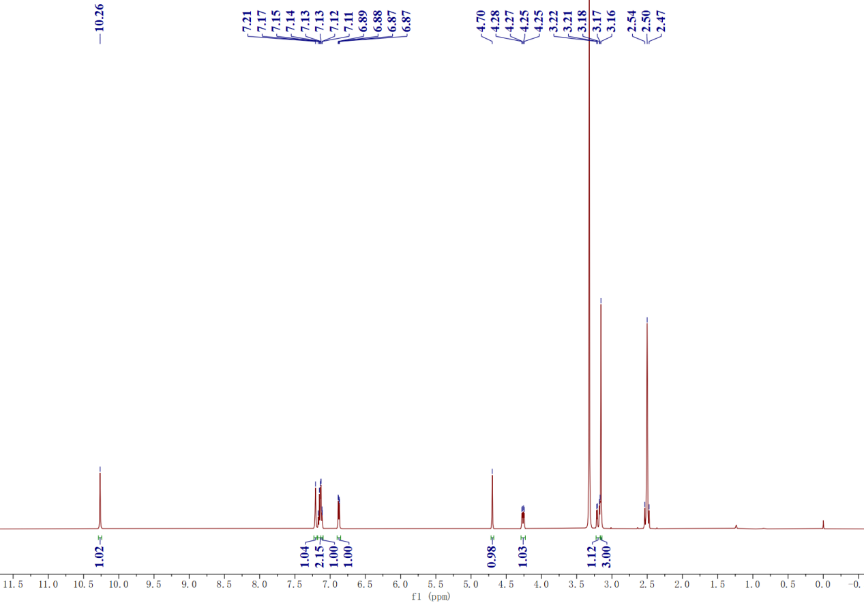


**Figure S17.** ^1^H NMR spectrum of compound **2** in DMSO-*d*_6_

_
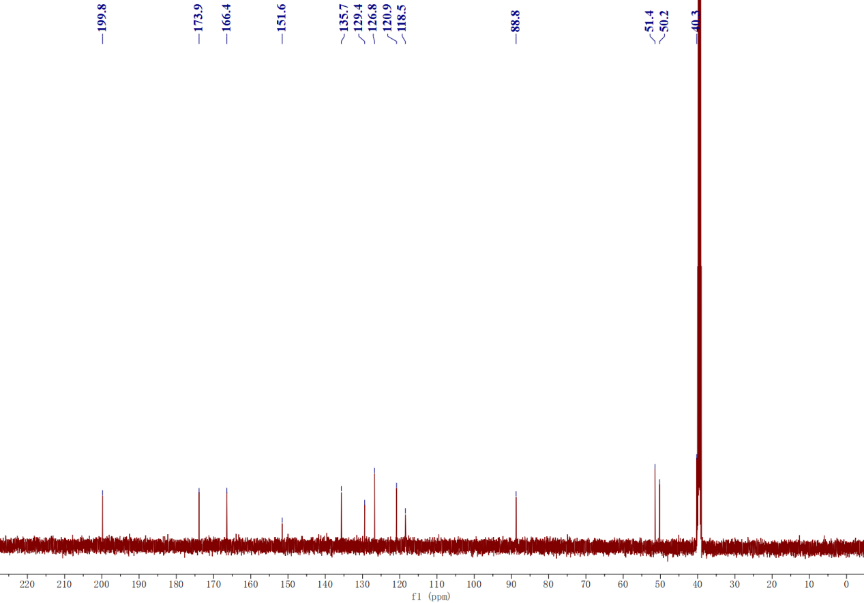
_

**Figure S18.** ^13^C NMR spectrum of compound **2** in DMSO-*d*_6_

_
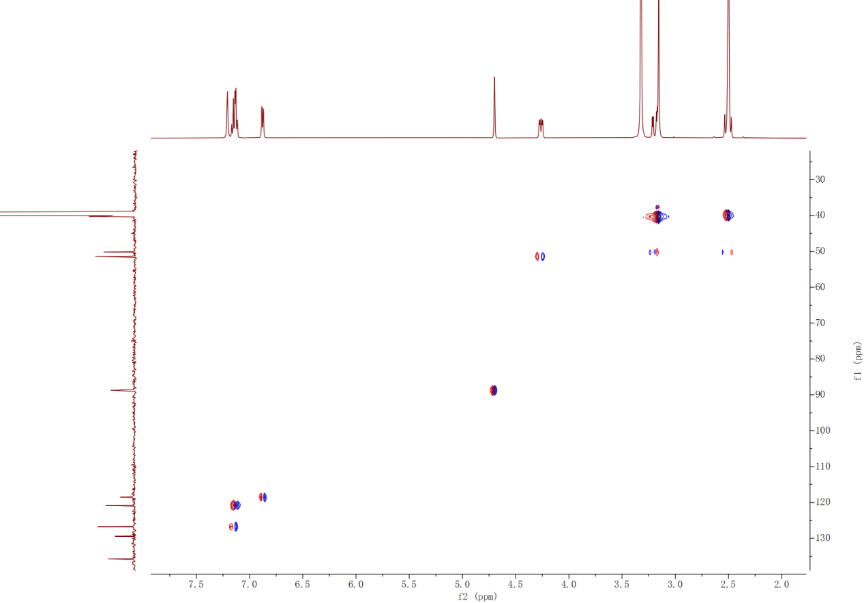
_

**Figure S19.** HSQC spectrum of compound **2** in DMSO-*d*_6_

_
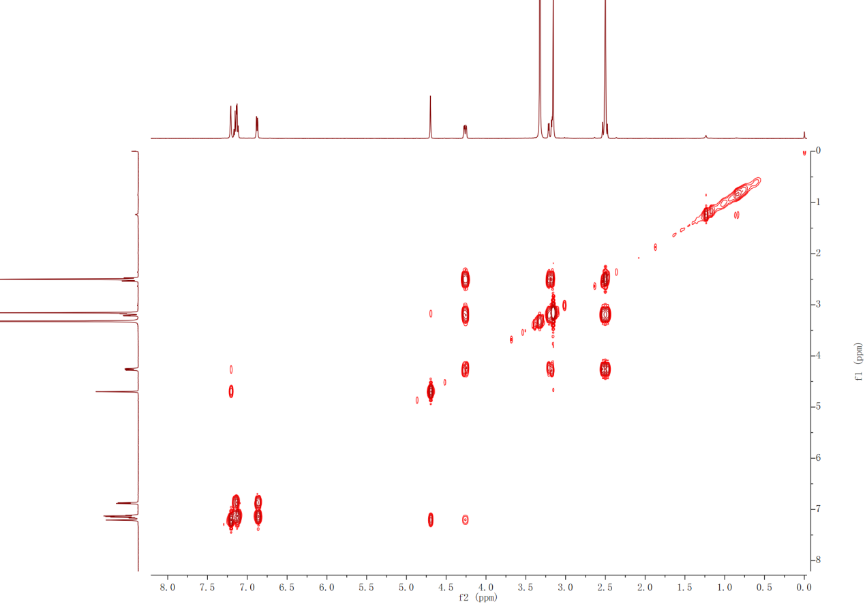
_

**Figure S20.** COSY spectrum of compound **2** in DMSO-*d*_6_

_
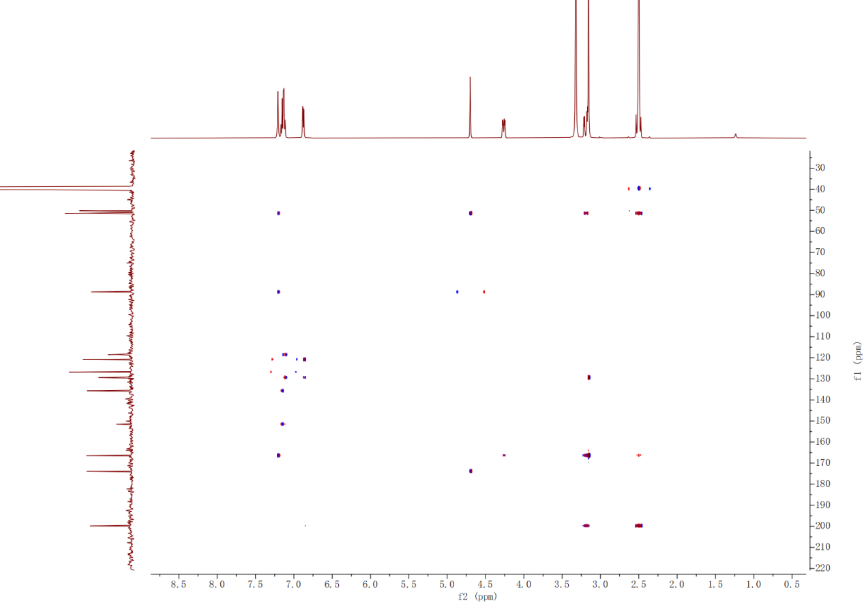
_

**Figure S21.** HMBC spectrum of compound **2** in DMSO-*d*_6_

_
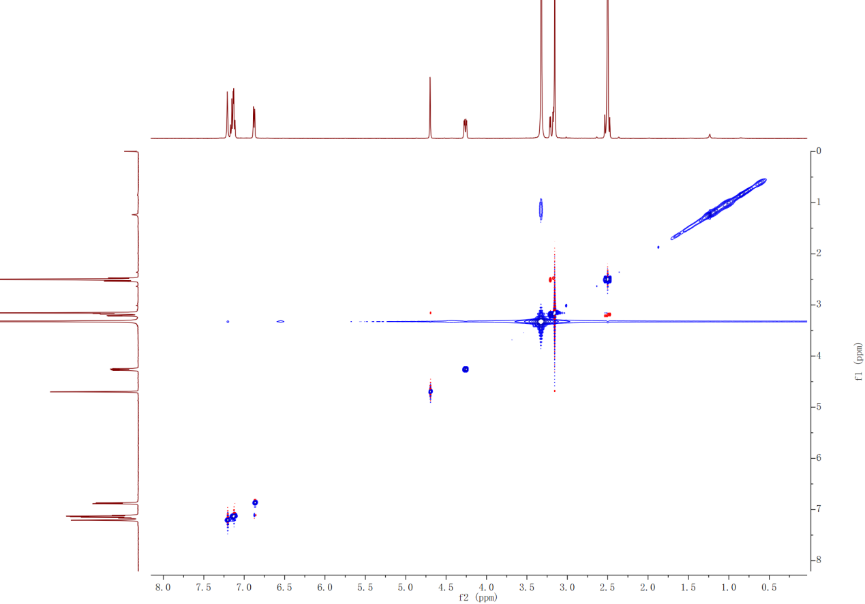
_

**Figure S22.** NOESY spectrum of compound **2** in DMSO-*d*_6_


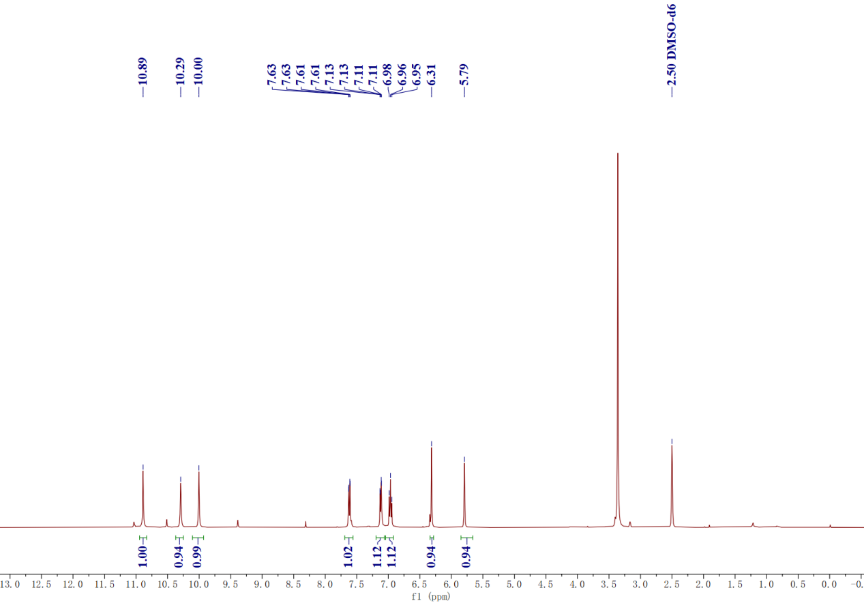


**Figure S23.** ^1^H NMR spectrum of compound **3** in DMSO-*d*_6._

_
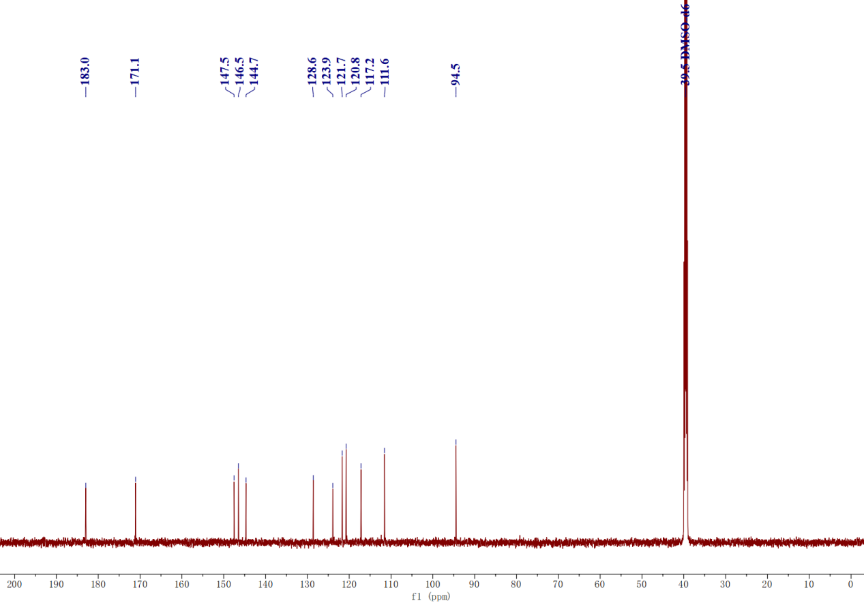
_**Figure S24.**^13^C NMR spectrum of compound **3** in DMSO-*d*_6._

1. **Supplementary Note 2**

**2.1. Total Synthesis**

**2.1.1. General methods**

All reactions were performed under nitrogen or argon in vacuum-dried glassware using dry solvents and standard syringe techniques. Reagents were purchased at the highest commercial quality (>95%) and used without further purification, unless otherwise stated. Anhydrous tetrahydrofuran (THF) was distilled from sodium-benzophenone, dichloromethane (CH_2_Cl_2_) was distilled from calcium hydride. Column chromatography was carried out by using silica gel (200−300 mesh). Yields refer to chromatographically, unless otherwise specified. Optical rotations were measured on a JASCO P-1020 digital polarimeter (JASCO Ltd., Tokyo, Japan). ECD spectra were acquired on a JASCO J-715 (JASCO) or Chirascan CD (Applied Photophysics) spectropolarimeter. IR spectra were obtained on a Nicolet Nexus 470 spectrophotometer (Perkin Elmer Ltd., Boston, MA, USA) in KBr discs. NMR spectra were recorded on a JEOL JEM-ECP NMR spectrometer (JEOL Ltd., Tokyo, Japan; 500 MHz for ^1^H and 125 MHz for ^13^C), using TMS as an internal standard. HRESIMS spectra were performed on a Thermo Scientific LTQ Orbitrap XL spectrometer. Single-crystal data were obtained on an Agilent Gemini Ultra diffractometer (Cu Kα radiation) (Agilent Technologies Inc., California, America). HPLC analysis was performed on a Hitachi L-2000 system (Hitachi Ltd., Tokyo, Japan) using a C18 column [(YMC Co., Ltd., Tokyo, Japan) YMC-Park, ODS-A, 250 × 4.6 mm, S-5 *μ*m, 12 nm, 1.0 mL/min]. Semi-preparative HPLC was performed on a Hitachi L-2000 system (Hitachi Ltd., Tokyo, Japan) using a C18 column [(Eka Ltd., Bohus, Sweden) Kromasil 250 × 10 mm, 5 *μ*m, 2.0 mL/min]. Racemic mixtures were resolved on Chiralpak IC column (5 *μ*m, 4.6 × 150 mm, hexane−ethanol eluent, 0.6 mL/min) and Chiralpak IA column (5 *μ*m, 4.6 × 250 mm, hexane−ethanol eluent, 0.6 mL/min). Silica gel ((Qingdao Haiyang Chemical Group Co., Qingdao, China; 200–300 mesh), octadecylsilyl silica gel (YMC Co., Ltd., Tokyo, Japan; 45−60 *μ*m), macroporous resin (H&E Co., Ltd., Connecticut, America) and Sephadex LH-20 (GE Ltd., Connecticut, America) were used for column chromatography. Precoated silica gel plates (Yantai Zhifu Chemical Group Co., Yantai, China; G60, F-254) were used for thin layer chromatography.

**2.1.2. Experimental procedures and characterizations**

**Preparation of Compound 10:**

A solution of L-aspartic acid diethyl ester hydrochloride **8** (34.16 g, 151.38 mmol) in dry dichloromethane (200 mL) was cooled to 0℃. Then, dry DIPEA (19.57 g, 151.38 mmol), monoethyl malonate **9** (20.0 g, 151.38 mmol), and 4-dimethylaminopyridine (5.55 g, 45.41 mmol) were sequentially added. After 0.5 h, 1-ethyl-3-(3-dimethylaminopropyl) carbodiimide hydrochloride (EDCI) (29.02 g, 151.38 mmol) and dry dichloromethane (100 mL) were added, and the mixture was stirred at room temperature for 12 h. After completion, the reaction was quenched with water (300 mL), and the aqueous layer was extracted with CH_2_Cl_2_ (3×200 mL). The combined organic layers were washed successively with water (500 mL) and brine (500 mL), dried over anhydrous Na_2_SO_4_ and evaporated under reduced pressure. The residue was purified by flash chromatography on silica gel (eluent: EtOAc/PE, 1/5 to 1/4) to give compound **10** (41.5 g, yield: 90%) as a yellow oil.

Compound **10**: **R_f_** 0.3 (3: 1 petroleum ether: ethyl acetate); [α]^29^_D_ –2 (*c* 0.6, MeOH). **IR** (neat, cm^–1^) ν_max_ 2985, 1741, 1683, 1533, 1373, 1349, 1285, 1200, 1030 cm^–1^; **^1^H NMR** (500 MHz, CDCl_3_) *δ* 7.82 (d, *J* = 8.1 Hz, 1H), 4.80 – 4.69 (m, 1H), 4.09 – 4.02 (overlapped, 6H), 3.24 (d, *J* = 1.8 Hz, 2H), 2.88 (dd, *J* = 17.0, 4.7 Hz, 1H), 2.73 (dd, *J* = 17.0, 4.8 Hz, 1H), 1.35 – 0.97 (dt, *J* = 13.4, 7.1 Hz, 9H). **^13^C NMR** (125 MHz, CDCl_3_) *δ* 170.5, 170.2, 168.4, 165.0, 61.6, 61.3, 60.8, 48.6, 41.3, 36.1, 13.9, 13.9, 13.8. **HRMS (ESI)**: m/z calcd for C_13_H_22_O_7_N [M+H]^+^ : 304.1391; found: 304.1388.

**Preparation of Compound 7:**

To a stirred solution of compound **10** (30.0 g, 98.91 mmol) in THF (200 mL) at -20 ℃ was added dropwise of a 1 mol•L^-1^ lithium bis(trimethylsilyl)amide (297 mL, 296.73 mmol) solution, and stirred at room temperature for 6 h. After completion, the organic phase was removed, and the aqueous phase was acidified to pH = 4. The mixture was filtered and washed with ethanol to afford **7** (17.8 g, yield: 70%) as a white solid.

Compound **7**: **R_f_** 0.2 (10: 1 CH_2_Cl_2_: MeOH); **IR** (neat, cm^–1^) ν_max_ 3290, 2982, 1731, 1686, 1634, 1478, 1374, 1258, 1227, 1174, 1097, 1064, 1027, 650 cm^–1^; **^1^H NMR** (400 MHz, DMSO-*d*_6_) *δ* 6.76 (s, 1H), 4.13 – 3.98 (m, 4H), 3.66 (ddd, *J* = 9.0, 4.2, 1.6 Hz, 1H), 2.60 (dd, *J* = 15.7, 4.1 Hz, 1H), 2.17 (dd, *J* = 15.7, 9.0 Hz, 1H), 1.91 (s, 1H), 1.18 (dt, *J* = 8.2, 7.1 Hz, 6H). **^13^C NMR** (100 MHz, DMSO-*d*_6_) *δ* 191.9, 172.0, 171.2, 167.3, 87.5, 60.0, 57.7, 56.0, 39.0, 14.7, 14.1. **HRMS (ESI)**: m/z calcd for C_11_H_16_O_6_N [M+H]^+^ : 258.0972; found: 258.0966.

**Preparation of Compound 11:**

To a solution of compound **7** (17.0 g, 48.5 mmol) in MeCN (400 mL) was added TFA (40.0 mL). The mixture was allowed to react at 100 ℃ for 8 h. The solvent was evaporated under reduced pressure to yield product **6** and the crude material was used in the next reaction without purification.

To a stirred solution of crude **6** in EtOH (200 mL) was added AcOH (10.0 mL) and o-Anisidine (24.2 g, 198.29 mmol), and the mixture was heated and stirred at 70 °C for 12 h. After completion, the organic solvent was removed in vacuo. The residue was purified by flash chromatography on silica gel (eluent: EtOAc/PE, 3/1 to 5/1) to give compound **11** (5.8 g, yield: 30%) as a yellow oil.

Compound **11**: **R_f_** 0.5 (10: 1 CH_2_Cl_2_: MeOH); **IR** (neat, cm^–1^) ν_max_ 3291, 2983, 1721, 1668, 1622, 1591, 1544, 1409, 1375, 1242, 1207, 1027, 801, 752 cm^–1^; **^1^H NMR** (500 MHz, DMSO-*d*_6_) *δ* 8.22 (s, 1H), 7.26 (d, *J* = 7.8 Hz, 1H), 7.20 (s, 1H), 7.02 (q, *J* = 7.6 Hz, 2H), 6.96 – 6.90 (m, 1H), 5.01 (s, 1H), 4.47 (dd, *J* = 7.9, 3.9 Hz, 1H), 4.11 (q, *J* = 7.1 Hz, 2H), 3.83 (s, 3H), 2.98 (dd, *J* = 16.2, 4.2 Hz, 1H), 2.44 (dd, *J* = 16.2, 8.2 Hz, 1H), 1.21 (t, *J* = 7.1 Hz, 3H).**^13^C NMR** (125 MHz, DMSO-*d*_6_) *δ* 174.5, 170.7, 160.0, 149.6, 129.9, 123.2, 120.7, 119.2, 111.5, 91.6, 60.3, 55.7, 53.9, 40.0, 14.0; **HRMS (ESI)**: m/z calcd for C_15_H_19_O_4_N_2_ [M+H]^+^ : 291.1339; found: 291.1334.

**Preparation of Compound 4:**

To a stirred solution of compound **11** (2.00 g, 6.89 mmol) in MeOH/H_2_O (1:1, 100 mL) was added LiOH (247 mg, 10.33 mmol), and the mixture was stirred at room temperature for 2 h. After completion, the organic solvent was removed in vacuo and the resulting mixture was diluted with H_2_O, acidified with 2 N HCl. The mixture was filtered and washed with ethanol to afford **4** (1.54 g, yield: 85%) as a white solid.

Compound **4**: **R_f_** 0.3 (3: 1 CH_2_Cl_2_: MeOH); **IR** (neat, cm^–1^) ν_max_ 3274, 1590, 1543, 1462, 1296, 1241, 1178, 1118, 1028, 794, 749, 688 cm^–1^; **^1^H NMR** (500 MHz, DMSO-*d*_6_) *δ* 10.47 (s, 1H), 7.25 (d, *J* = 7.2 Hz, 1H), 7.00 – 6.95 (m, 2H), 6.95 – 6.87 (m, 2H), 4.98 (s, 1H), 4.35 (d, *J* = 8.1 Hz, 1H), 3.78 (s, 3H), 2.49 (overlapped, 1H), 2.22 (dd, *J* = 16.5, 10.4 Hz, 1H). **^13^C NMR** (125 MHz, DMSO-*d*_6_) *δ* 174.7, 173.6, 162.4, 149.4, 130.8, 122.1, 120.6, 117.7, 111.3, 89. 3, 55.7, 55.4, 42.9; **HRMS (ESI)**: m/z calcd for C_13_H_15_O_4_N_2_ [M+H]^+^ : 263.1026; found:263.1020.

**Preparation of Compound 12:**

Heating of compound **4** (1.2 g, 4.58 mmol) in polyphosphoricacid (PPA) at 120 ℃ for 1 h directly led to **12**. After completion, the mixture was diluted with H_2_O (100 mL), then the solution was washed with excess of saturated NaHCO_3_ (4 × 100 mL) and finally with brine (100 mL). The solvent was removed under reduced pressure and purified by flash chromatography on silica gel (eluent: CH_2_Cl_2_/MeOH, 20/1 to 10/1) to give compound **12** (251 mg, yield: 22%) as a yellow oil.

Compound **12**: **R_f_** 0.4 (10: 1 CH_2_Cl_2_: MeOH); **IR** (neat, cm^–1^) ν_max_ 3252, 1669, 1627, 1573, 1491, 1463, 1381, 1259, 1245, 1208, 1037 cm^–1^; **^1^H NMR** (400 MHz, DMSO-*d*_6_) *δ* 9.27 (s, 1H), 7.38 (dd, *J* = 8.2, 1.4 Hz, 1H), 7.27 (s, 1H), 7.24 (dd, *J* = 8.0, 1.5 Hz, 1H), 7.02 (t, *J* = 8.0 Hz, 1H), 5.13 (d, *J* = 1.7 Hz, 1H), 4.53 (dd, *J* = 13.2, 2.4 Hz, 1H), 3.90 (s, 3H), 3.06 (dd, *J* = 17.4, 3.2 Hz, 1H), 2.85 (dd, *J* = 17.4, 13.1 Hz, 1H).**^13^C NMR** (100 MHz, DMSO-*d*_6_) *δ* 195.1, 173.8, 162.7, 149.2, 130.3, 125.5, 122.1, 121.5, 115.3, 93.4, 56.5, 50.2, 47.1; **HRMS (ESI)**: m/z calcd for C_13_H_13_O_3_N_2_ [M+H]^+^ : 245.0921; found: 245.0918.

**Preparation of asperazepanone A (1):**

To a stirring solution of compound **12** (30 mg, 122.82 *μ*mol) in dry CH_2_Cl_2_ (2 mL), BBr_3_ was added dropwise at room temperature and stirred for 2 h. After completion, the reaction was quenched by pouring on ice. The solvent was removed under reduced pressure and the residue was purified by flash chromatography on silica gel (eluent: CH_2_Cl_2_/MeOH, 10/1 to 5/1) to give asperazepanone A (**1**) (21 mg, yield: 74%) as a yellow soild.

Asperazepanone A (**1**): **R_f_** 0.2 (10: 1 CH_2_Cl_2_: MeOH); **^1^H NMR** (400 MHz, DMSO-*d*_6_) *δ* 10.47 (s, 1H), 9.13 (s, 1H), 7.27 (dd, *J* = 8.1, 1.5 Hz, 1H), 7.23 (d, *J* = 2.1 Hz, 1H), 7.06 (dd, *J* = 7.7, 1.6 Hz, 1H), 6.87 (t, *J* = 7.9 Hz, 1H), 5.13 (s, 1H), 4.51 (d, *J* = 11.0 Hz, 1H), 3.04 (dd, *J* = 17.3, 3.0 Hz, 1H), 2.85 (dd, *J* = 17.2, 13.0 Hz, 1H). **^13^C NMR** (150 MHz, DMSO-*d*_6_) *δ* 195.1, 173.9, 162.8, 147.3, 129.5, 125.6, 121.5, 120.9, 118.7, 92.9, 50.2, 47.0; **HRMS (ESI)**: m/z calcd for C_12_H_11_O_3_N_2_ [M+H]^+^ : 231.0764; found: 231.0762.

**Preparation of asperazepanone B (2):**

To a solution of compound **12** (40 mg, 163.77 umol) in acetone (2 mL) was added Cs_2_CO_3_ (106.72 mg, 327.53 umol) and CH_3_I (34.87 mg, 245.65 *μ*mol). After completion, the solvent was evaporated under reduced pressure to yield product **13** and the crude material was used in the next reaction without purification.

To a stirring solution of compound **13** in dry CH_2_Cl_2_ (2 mL), BBr_3_ was added dropwise at room temperature and stirred for 2 h. After completion, the reaction was quenched by pouring on ice. The solvent was removed under reduced pressure and the residue was purified by flash chromatography on silica gel (eluent: CH_2_Cl_2_/MeOH, 10/1 to 5/1) to give asperazepanone B (**2**) (27 mg, yield: 68%) as a yellow soild.

Asperazepanone B (**2**): **R_f_** 0.25 (10: 1 CH_2_Cl_2_: MeOH); **^1^H NMR** (600 MHz, DMSO-*d*_6_) *δ* 7.22 (s, 1H), 7.17 – 7.13 (m, 1H), 7.12 (dd, *J* = 8.1, 1.8 Hz, 1H), 6.88 – 6.84 (m, 1H), 4.70 (s, 1H), 4.28 (dd, *J* = 12.4, 5.0 Hz, 1H), 3.23 – 3.19 (m, 1H), 3.18 (s, 3H), 2.51(overlapped, 1H). **^13^C NMR** (150 MHz, DMSO-*d*_6_) *δ* 199.8, 173.9, 166.4, 151.6, 135.7, 129.4, 126.7, 120.8, 118.4, 88.7, 51.4, 50.2, 40.2. **HRMS (ESI)**: m/z calcd for C_13_H_13_O_3_N_2_ [M+H]^+^ : 245.0921; found: 245.0917

Asperazepanone B (**2**) (10.0 mg) was resolved into the corresponding pure enantiomers (+)-**2** (*t*_R_ = 58.0 min, 4.0 mg), (−)-**2** (*t*_R_ = 63.6 min, 4.0 mg) by HPLC using a Chiralpak IC chiral-phase column [5 *μ*m, 4.6 × 150 mm, hexane−ethanol eluent (80:20), 0.6 mL/min].

(−)**-**Asperazepanone B [(−)-10a*S*-**2**]: yellow needle; [α]^25^_D_ −130 (*c* 0.2, MeOH).

**2.1.3. Comparison of the ^1^H NMR and ^13^C NMR Spectra of Isolated and Synthetic Compounds**


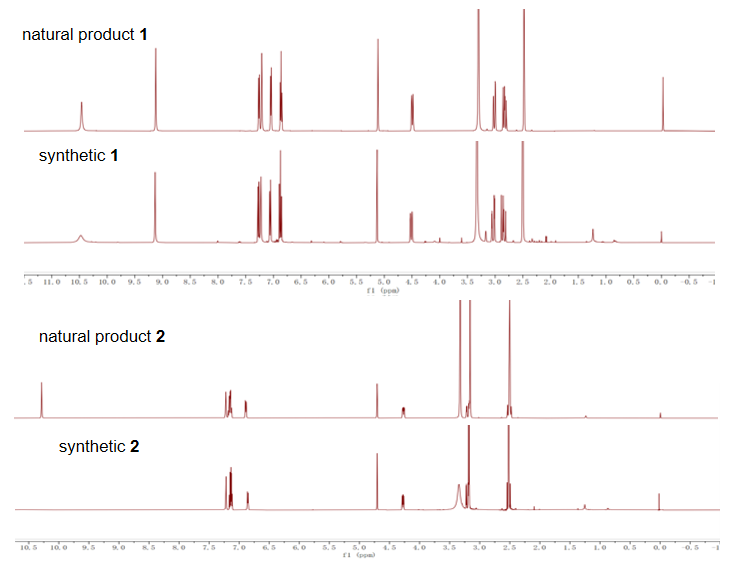


**Figure S25.** Comparison of ^1^H NMR spectra of natural product **1** and **2** with synthetic **1** and **2**.


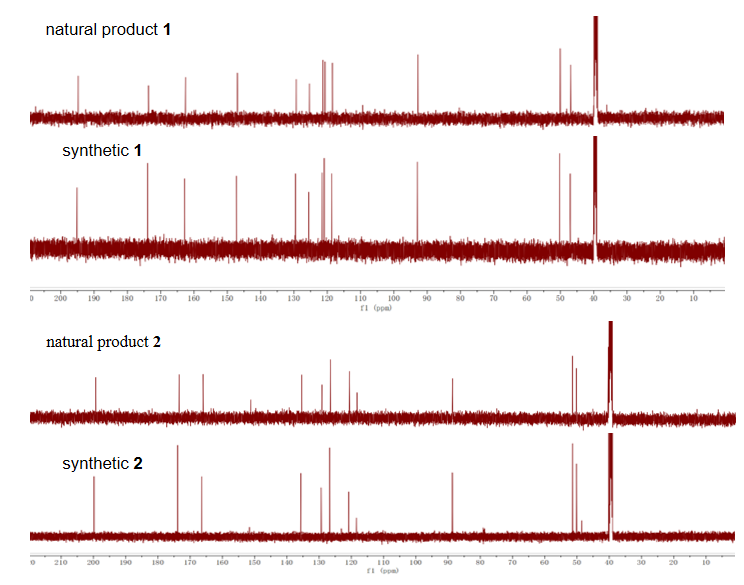


**Figure S26**. Comparison of ^13^C NMR spectra of natural products **1** and **2** with synthetic **1** and **2**.

**Table S2.** ^1^H NMR and ^13^C NMR Data comparison of natural product **1** and synthetic **1**.

| ^1^H NMR Data | Synthetic  (600 MHz) | Natural  (500 MHz) | ^13^C NMR Data | Synthetic  (150 MHz) | Natural  (125 MHz) |
| --- | --- | --- | --- | --- | --- |
| Position | *δ*_H_ (*J* in Hz) | | Position | *δ*_C_ | |
| 1 | 7.23, 1H, s | 7.22, 1H, s | 1 |  |  |
| 2 |  |  | 2 | 173.9 | 173.9 |
| 3 | 5.13, 1H, s | 5.13, 1H, s | 3 | 92.9 | 92.9 |
| 3a |  |  | 3a | 162.8 | 162.8 |
| 4a |  |  | 4a | 129.5 | 129.5 |
| 5 |  |  | 5 | 147.3 | 147.2 |
| 6 | 7.06, 1H, dd, 7.7, 1.6 | 7.06, 1H, dd, 7.9, 1.2 | 6 | 118.7 | 118.6 |
| 7 | 6.87, 1H, t, 7.9 | 6.87, 1H, t, 7.9 | 7 | 121.5 | 121.5 |
| 8 | 7.27, 1H, dd, 8.1, 1.6 | 7.27, 1H, dd, 7.9, 1.2 | 8 | 120.9 | 120.9 |
| 8a |  |  | 8a | 125.6 | 125.5 |
| 9 |  |  | 9 | 195.1 | 195.1 |
| 10 | 2.85, 1H, dd, 17.2, 13.0 | 2.85, 1H, dd, 17.2, 13.1 | 10 | 47.0 | 47.0 |
|  | 3.04, 1H, dd, 17.3, 3.0 | 3.03, 1H, dd, 17.2, 2.8 |  |  |  |
| 10a | 4.51, 1H, dd, 13.1, 2.8 | 4.51, 1H, dd, 13.1, 2.8 | 10a | 50.2 | 50.2 |
| 4-NH | 9.13, 1H, s | 9.13, 1H, s | 4-NH |  |  |
| 5-OH | 10.47, 1H, s | 10.46, 1H, s | 5-OH |  |  |

^a^ Measured in DMSO-*d*_6_.

**Table S3.** ^1^H NMR and ^13^C NMR Data comparison of natural product **2** and synthetic **2**.

| ^1^H NMR Data | Synthetic  (600 MHz) | Natural  (500 MHz) | ^13^C NMR Data | Synthetic  (150 MHz) | Natural  (125 MHz) |
| --- | --- | --- | --- | --- | --- |
| Position | *δ*_H_ (*J* in Hz) | | Position | *δ*_C_ | |
| 1 | 7.20, 1H, s | 7.21, 1H, s | 1 |  |  |
| 2 |  |  | 2 | 173.9 | 173.9 |
| 3 | 4.68, 1H, s | 4.70, 1H, s | 3 | 88.7 | 88.8 |
| 3a |  |  | 3a | 166.4 | 166.4 |
| 4a |  |  | 4a | 135.7 | 135.7 |
| 5 |  |  | 5 | 151.6 | 151.6 |
| 6 | 7.13, 1H, overlapped | 7.15, 1H, overlapped | 6 | 126.7 | 126.8 |
| 7 | 7.10, 1H, overlapped | 7.12, 1H, overlapped | 7 | 120.8 | 120.9 |
| 8 | 6.84, 1H, dd (7.2, 1.9) | 6.88, 1H, dd (7.2, 1.6) | 8 | 118.4 | 118.5 |
| 8a |  |  | 8a | 129.4 | 129.4 |
| 9 |  |  | 9 | 199.8 | 199.8 |
| 10 | 2.52, 1H, overlapped | 2.50, dd (19.0, 11.9) | 10 | 50.2 | 50.2 |
|  | 3.18, 1H, overlapped | 3.19, dd (19.0, 4.3) |  |  |  |
| 10a | 4.28, 1H, dd (12.2, 4.7) | 4.26, 1H, dd (11.9, 4.3) | 10a | 51.4 | 51.4 |
| 4-NH |  |  | 4-NH |  |  |
| 5-OH |  | 10.26, 1H, s | 5-OH |  |  |
| 4-CH_3_ | 3.16, 1H, s | 3.16, 1H, s | 4-CH_3_ | 40.2 | 40.3 |

^a^ Measured in DMSO-*d*_6_.

**2.1.4. MS data, IR spectrum, Chiral-phase HPLC analysis and Experimental ECD spectra.**

**Figure S27.** HRESIMS spectrum of compound **10**.


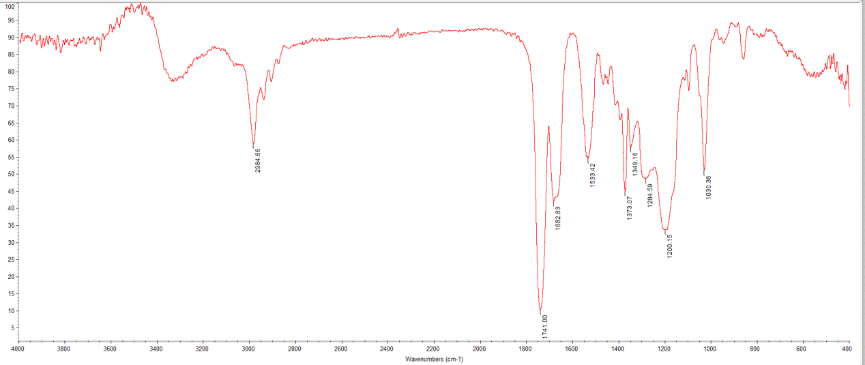


**Figure S28.** IR spectrum of compound **10**.

**Figure S29.** HRESIMS spectrum of compound **7**.


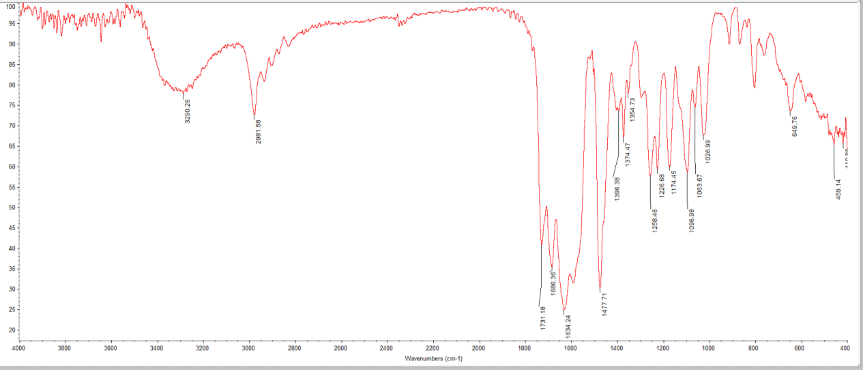


**Figure S30.** IR spectrum of compound **7**.

**Figure S31.** HRESIMS spectrum of compound **11**.


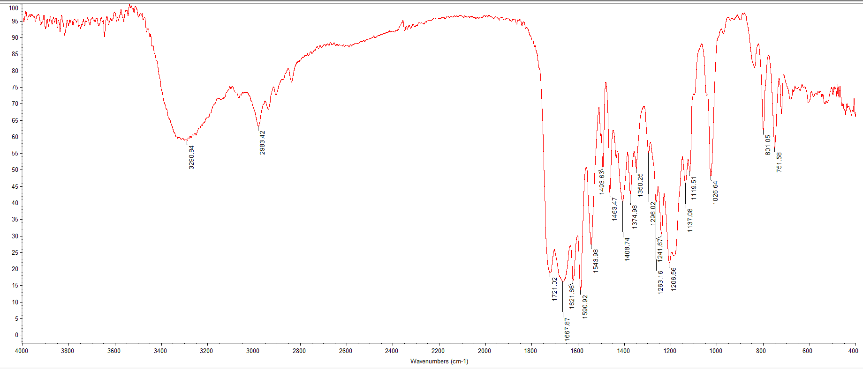


**Figure S32.** IR spectrum of compound **11**.

**Figure S33.** HRESIMS spectrum of compound **4**.


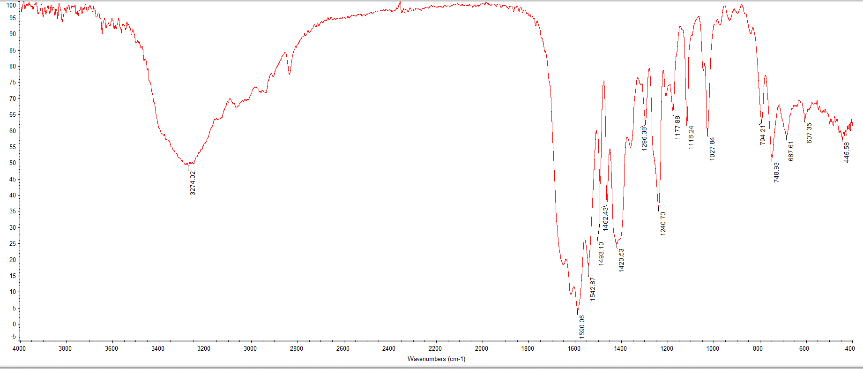


**Figure S34.** IR spectrum of compound **4**.

**Figure S35.** HRESIMS spectrum of compound **12**.


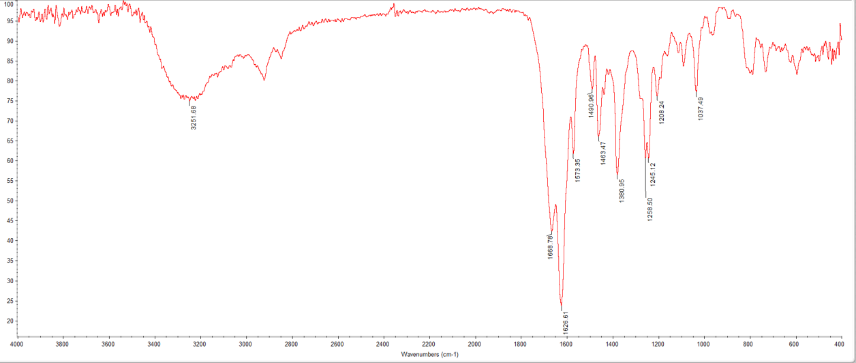
**Figure S36.** IR spectrum of compound **12**.

**Figure S37.** HRESIMS spectrum of total synthesis compound **1**.

**Figure S38.** HRESIMS spectrum of total synthesis compound **2**.


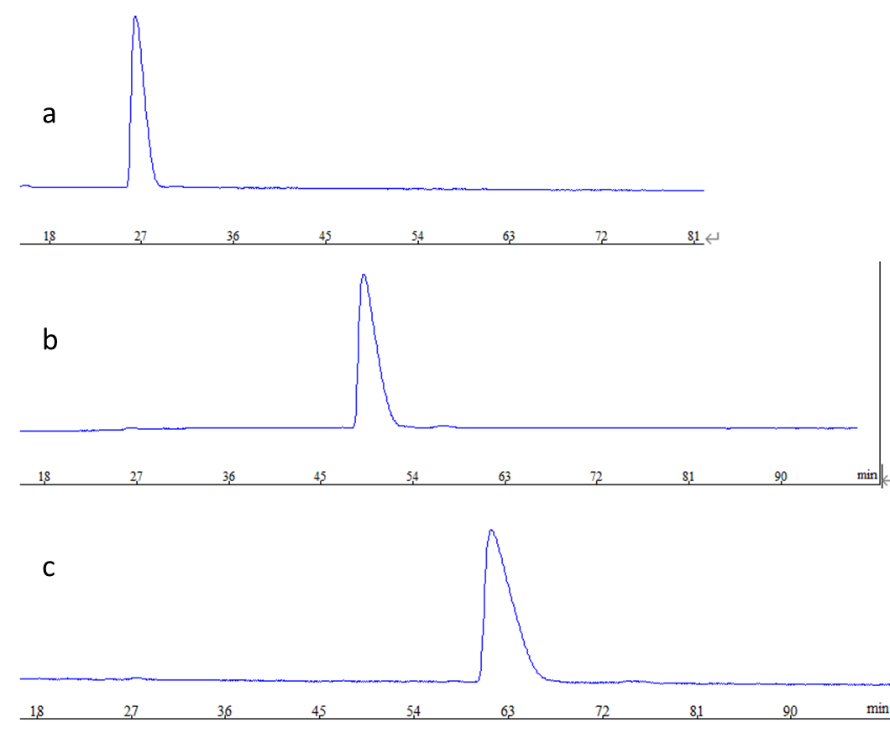


**Figure S39.** Chromatogram of the chiral-phase HPLC of compound **10**. (**a**) Compound **10** was analyzed by HPLC using a Chiralpak IC chiral-phase column [5 *μ*m, 4.6 × 150 mm, hexane−ethanol eluent (80: 20), 0.6 mL/min]. (**b**) Compound **10** was analyzed by HPLC using a Chiralpak IC chiral-phase column [5 *μ*m, 4.6 × 150 mm, hexane−ethanol eluent (85: 15), 0.6 mL/min]. (**c**) Compound **10** was analyzed by HPLC using a Chiralpak IC chiral-phase column [5 *μ*m, 4.6 × 150 mm, hexane−ethanol eluent (90: 10), 0.6 mL/min].


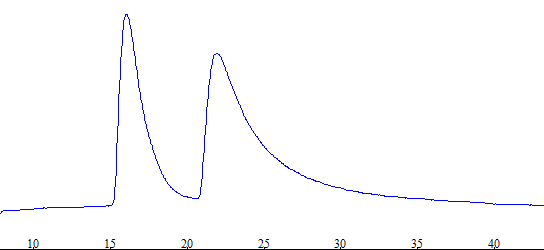


**Figure S40.** Chromatogram of the chiral-phase HPLC of compound **7**. Compound **7** was analyzed by HPLC using a Chiralpak IA chiral-phase column [5 *μ*m, 4.6 × 250 mm, hexane−ethanol eluent (70: 30), 0.6 mL/min].


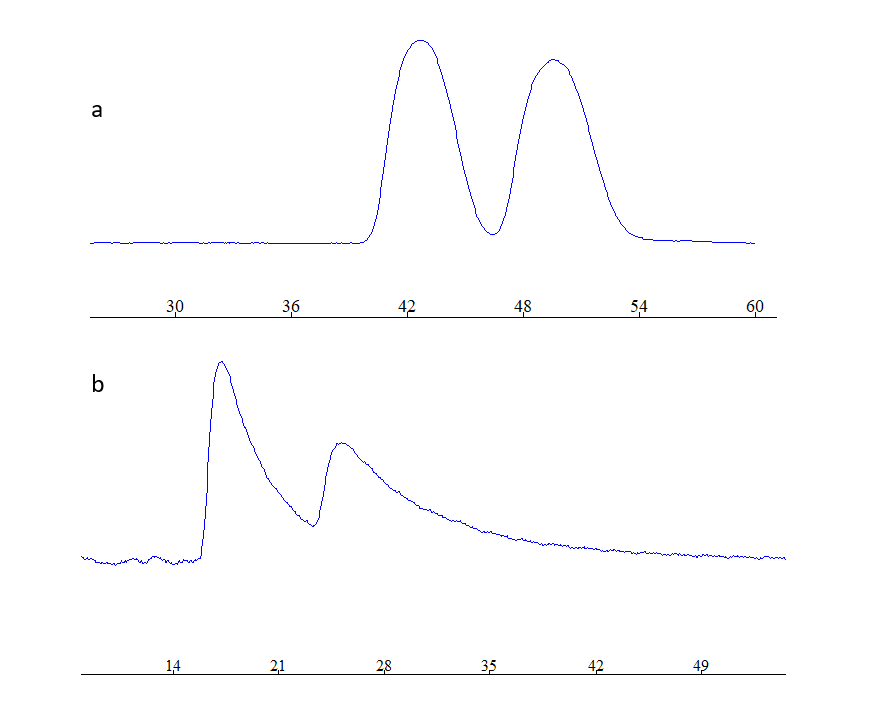


**Figure S41.** (**a**) Chromatogram of the chiral-phase HPLC of compound **11**. Compound **11** was analyzed by HPLC using a Chiralpak IC chiral-phase column [5 *μ*m, 4.6 × 150 mm, hexane−ethanol eluent (80: 20), 0.6 mL/min].

(**b**) Chromatogram of the chiral-phase HPLC of compound **4**. Compound **4** was analyzed by HPLC using a Chiralpak IC chiral-phase column [5 *μ*m, 4.6 × 150 mm, hexane−ethanol eluent (80: 20), 0.6 mL/min].

**Figure S42.** Chromatogram of the chiral-phase HPLC of compound **12**. Compound **12** was analyzed by HPLC using a Chiralpak IC chiral-phase column [5 *μ*m, 4.6 × 150 mm, hexane−ethanol eluent (60: 40), 0.6 mL/min].

**Figure S43.** Chromatogram of the chiral-phase HPLC of synthesized compound **1**. Compound **1** was analyzed by HPLC using a Chiralpak IC chiral-phase column [5 *μ*m, 4.6 × 150 mm, hexane−ethanol eluent (70: 30), 0.6 mL/min].


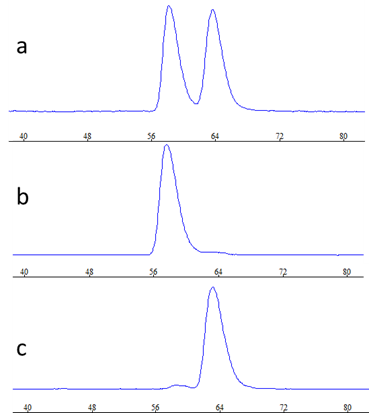


**Figure S44.** (**a**) Chromatogram of the chiral-phase HPLC of synthesized compound **2**. (**b**) Chromatogram of the chiral-phase HPLC of synthesized (+)-**2**. (**c**) Chromatogram of the chiral-phase HPLC of synthesized (−)-**2**. Compound **2** was analyzed by HPLC using a Chiralpak IC chiral-phase column [5 *μ*m, 4.6 × 150 mm, hexane−ethanol eluent (80: 20), 0.6 mL/min].


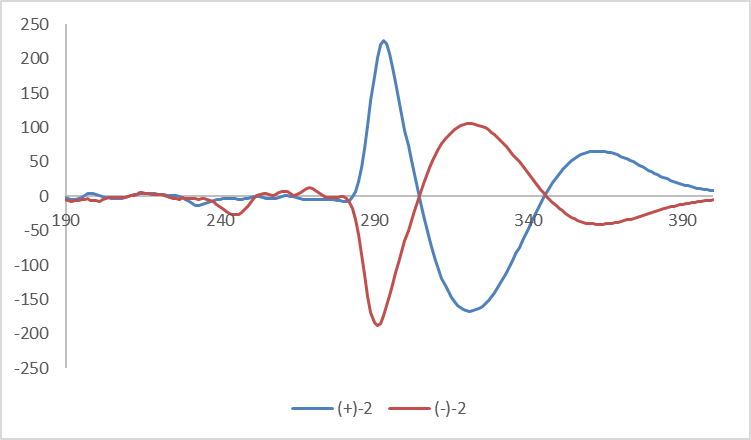


**Figure S45.** Experimental ECD spectra of (+)-**2** and (−)-**2**.

**2.1.5. NMR spectra**

**Figure S46.** ^1^H NMR spectrum of compound **10** in CDCl_3_.

**Figure S47.**^13^C NMR spectrum of compound **10** in CDCl_3._

**Figure S48.** ^1^H NMR spectrum of compound **7** in DMSO-*d*_6_.

**Figure S49.**^13^C NMR spectrum of compound **7** in DMSO-*d*_6._

**Figure S50.** ^1^H NMR spectrum of compound **11** in DMSO-*d*_6_.

**Figure S51.**^13^C NMR spectrum of compound **11** in DMSO-*d*_6._

**Figure S52.** ^1^H NMR spectrum of compound **4** in DMSO-*d*_6_.

**Figure S53.**^13^C NMR spectrum of compound **4** in DMSO-*d*_6._

**Figure S54.** ^1^H NMR spectrum of compound **12** in DMSO-*d*_6_.

**Figure S55.**^13^C NMR spectrum of compound **12** in DMSO-*d*_6._

**Figure S56.** ^1^H NMR spectrum of total synthesis compound **1** in DMSO-*d*_6_.

**Figure S57.**^13^C NMR spectrum of total synthesis compound **1** in DMSO-*d*_6._

**Figure S58.** ^1^H NMR spectrum of total synthesis compound **2** in DMSO-*d*_6_.

**Figure S59.**^13^C NMR spectrum of total synthesis compound **2** in DMSO-*d*_6._

**3. Supplementary Note 3**

**3.1. Bioactivity screening**

**3.1.1.** **Materials and reagents**

3-(4,5-Dimethylthiazol-2-yl)-2,5-diphenyltetrazolium bromide reagent (MTT), NG-Monomethyl-L-arginine, Monoacetate Salt (L-NMMA) were purchased from Beyotime Biotechnology Co.,Ltd (Shanghai, China). Mouse TNF-α ELISA kit (CT303A) and Mouse IL-6 ELISA kit (CT299A) were purchased from Dakewe Biotech Co.,Ltd (BeiJing, China). Nitric Oxide (NO) assay kit (A013-2-1) was purchased from Nanjing Jiancheng Bioengineering Institute (Nanjing, China).

**3.1.2.** **Cell viability and** **NO, TNF-α and IL-6 assay**

RAW264.7 cells were cultured with Dulbecco’s modified Eagle’s medium (DMEM), 10% FBS, 100 U/mL penicillin and 100 *µ*g/mL streptomycin in 37 ℃, 5% CO_2_ incubator. The cells were seeded in 96-well plates at 1 × 10^4^ cells/well for 12 h and supplemented with different concentrations of (+)-**2** for 24 h. Then, the MTT (working concentration: 0.5mg/ mL) mixed with DMEM was added to each well. After 4 h, the DMSO was added instead of the mix and the optical density was measured at 590 nm.

The levels of NO, TNF-α and IL-6 were detected by different kits. The cells were seeded in 24-well plates at the density of 4× 10^4^ cells/well for 24 h. Subsequently, cells were treated with with LPS (1 *μ*g/mL) and different concentrations of compound (+)-**2** for 24h. At the end of this time, the culture medium was collected to determine the concentration of NO, TNF-α and IL-6.

**3.1.3. Effect of compound (+)-2 on the viability and NO production of RAW264.7 cells**
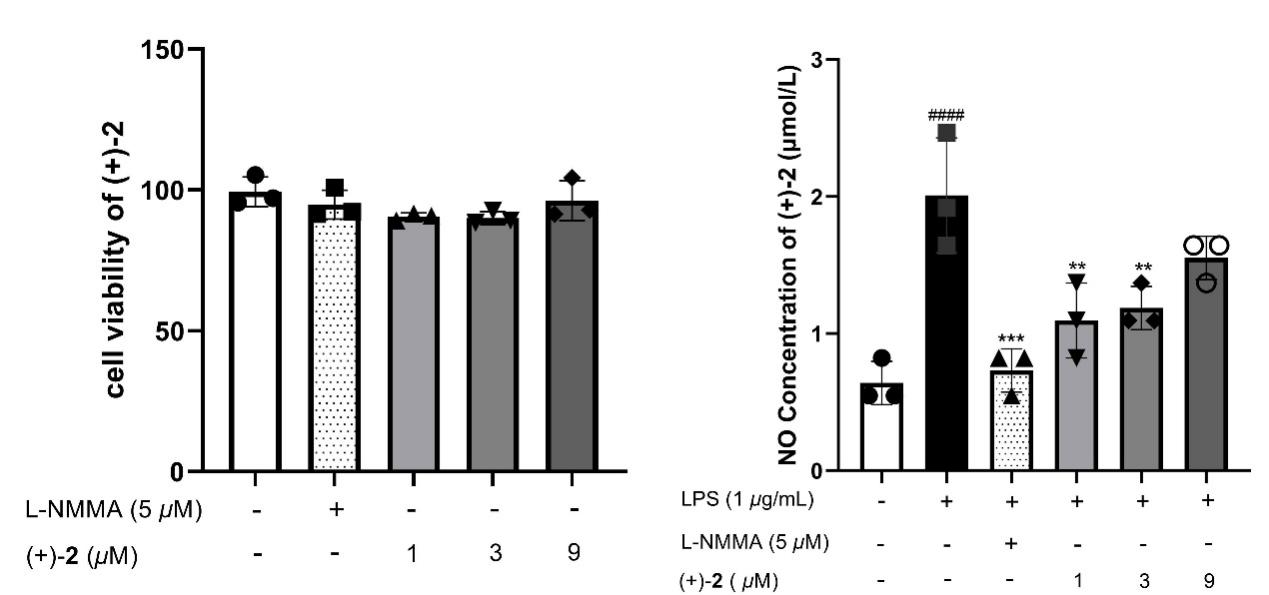


**Figure S60.** The cell viability of (+)-**2** in RAW 264.7 cells and effects of compound (+)-**2** on NO production by LPS stimulated RAW264.7 macrophages cells. MTT assay was used to test cell viability, the control group was defined as 100%. The medium was collected to detect NO concentration after that the cells were treated with the (+)-**2** (1, 3, 9 *μ*M) and LPS (1 *μ*g/mL) for 24 h. Data are presented as the mean of three experiments ± SD. ^####^p < 0.0001 compared to the control group; **p < 0.01, ***p < 0.001 compared to the LPS-treated group.

**4. Supplementary Reference**

1．Gao, W. T., Xing, X. D., Li, Y. & Lan, S. A novel construction of quino-fused tropone skeleton: first synthesis of 12*H*-benzo[4,5]cyclohepta[1,2-*b*]quinolin-12-one derivatives. *Tetrahedron* **70**, 2180–2189 (2014).
