# Supplementary material for: Discovery, total syntheses and potent anti-inflammatory activity of pyrrolinone-fused benzoazepine alkaloids Asperazepanones A and B from Aspergillus candidus
Source: Commun Chem. 2022 Jul 6;5:80. doi: 10.1038/s42004-022-00696-2 (PMC9814288; doi:10.1038/s42004-022-00696-2)
Supplement: Supplementary file 2 — Description of Additional Supplementary Files [file 42004_2022_696_MOESM2_ESM.pdf]

Description of Additional Supplementary Files

File Name: Supplementary Data 1

Description: X-ray of **1**

File Name: Supplementary Data 2

Description: X-ray of **2**
